# Cross-Continental Comparison of National Food Consumption Survey Methods—A Narrative Review

**DOI:** 10.3390/nu7053587

**Published:** 2015-05-13

**Authors:** Willem De Keyzer, Tatiana Bracke, Sarah A. McNaughton, Winsome Parnell, Alanna J. Moshfegh, Rosangela A. Pereira, Haeng-Shin Lee, Pieter van’t Veer, Stefaan De Henauw, Inge Huybrechts

**Affiliations:** 1 Faculty of Science and Technology, Department of Bio- and Food Sciences, University College Ghent-Campus Vesalius, Keramiekstraat 80, B-9000 Ghent, Belgium; E-Mails: bracketatiana@gmail.com (T.B.); stefaan.dehenauw@ugent.be (S.D.H.); 2 Department of Public Health, Ghent University University Hospital 4K3, De Pintelaan, 185, B-9000 Ghent, Belgium; E-Mail: HuybrechtsI@iarc.fr; 3 Centre for Physical Activity and Nutrition Research, School of Exercise and Nutrition Sciences, Deakin University, 221 Burwood Highway, Melbourne, Victoria 3125, Australia; E-Mail: sarah.mcnaughton@deakin.edu.au; 4 Division of Sciences, Department of Human Nutrition, University of Otago, PO Box 56, Dunedin 9054, New Zealand; E-Mail: winsome.parnell@otago.ac.nz; 5 Beltsville Human Nutrition Research Center, Agricultural Research Service–USDA, 10300 Baltimore Ave., Beltsville MD 20705, USA; E-Mail: Alanna.Moshfegh@ars.usda.gov; 6 Department of Social Nutrition, Instituto de Nutrição Josué de Castro, Federal University of Rio de Janeiro, Av. Carlos Chagas Filho 373, Cidade Universitária, Ilha do Fundão, Rio de Janeiro 21941-902, Brazil; E-Mail: rpereira@uol.com.br; 7 Nutrition Management Service and Policy Team, Korea Health Industry Development Institute, Chungbuk 363-700, Korea; E-Mail: haengsin@live.unc.edu; 8 Division of Human Nutrition, Wageningen University, Bomenweg 2, Wageningen 6703HD, The Netherlands; E-Mail: Pieter.vantVeer@wur.nl; 9 International Agency for Research on Cancer (IARC), 150 Cours Albert Thomas, 69372 Lyon Cedex 08, France

**Keywords:** nutrition surveys, public health surveillance, nutrition assessment, adult

## Abstract

Food consumption surveys are performed in many countries. Comparison of results from those surveys across nations is difficult because of differences in methodological approaches. While consensus about the preferred methodology associated with national food consumption surveys is increasing, no inventory of methodological aspects across continents is available. The aims of the present review are (1) to develop a framework of key methodological elements related to national food consumption surveys, (2) to create an inventory of these properties of surveys performed in the continents North-America, South-America, Asia and Australasia, and (3) to discuss and compare these methodological properties cross-continentally. A literature search was performed using a fixed set of search terms in different databases. The inventory was completed with all accessible information from all retrieved publications and corresponding authors were requested to provide additional information where missing. Surveys from ten individual countries, originating from four continents are listed in the inventory. The results are presented according to six major aspects of food consumption surveys. The most common dietary intake assessment method used in food consumption surveys worldwide is the 24-HDR (24 h dietary recall), occasionally administered repeatedly, mostly using interview software. Only three countries have incorporated their national food consumption surveys into continuous national health and nutrition examination surveys.

## 1. Introduction

Food consumption surveys (FCS) are used to estimate intakes of foods and nutrients by a certain target population from a specified region. Usually, they are initiated by governmental organizations to (1) identify deficient or excessive intakes of nutrients, (2) assess accordance with food-based dietary guidelines, or (3) estimate food safety related risks (e.g., contaminant exposures), using national representative samples. However, in light of comparability of results cross-continentally, a thorough overview and comparison of methodological aspects associated with these surveys in each continent is requested and has therefore been initiated in this cross-continental comparison of national food consumption survey methods.

In Europe, efforts have been made to harmonize methodological aspects related to dietary intake assessment (DIA) in the context of national nutrition surveys. Briefly, in the European Food Consumption Survey Method project (EFCOSUM), it was agreed that two non-consecutive 24-HDR (24 h dietary recall), are the most suitable to get internationally comparable data on population means and distributions of actual intake [1]. In addition, the menu-driven standardized 24-HDR program EPIC-Soft (IARC, Lyon, France) was considered to be the most appropriate software for standardized data collection in a pan-European survey. Following the EFCOSUM project, in the European Food Consumption Validation (EFCOVAL) project, EPIC-Soft was upgraded and adapted, and the two non-consecutive 24-HDRs using EPIC-Soft were validated using urinary biomarkers [2]. The software was further evaluated for use in the European Union (EU) Menu project [3], a pan-European food consumption survey among EU member states led by EFSA via the feasibility studies EMP-PANEU (Food Consumption Data Collection Methodology for the EU Menu Survey) and PANCAKE (Pilot study for Assessment of Nutrient intake and food Consumption Among Kids in Europe) [4,5,6]. In 2014, an EFSA report was published aiming to identify and evaluate available European data collection protocols and tools for capturing food consumption information [7]. Previously, Huybrechts *et al*. reported on the experiences from European national or regional dietary monitoring surveys using the standardized EPIC-Soft program [8], making a further inventory on this standardized methodology used in Europe redundant and leading to the decision to exclude Europe from this cross-continental inventory. 

Within the framework of the African Study on Physical Activity and Dietary Assessment Methods (AS-PADAM) project, an inventory questionnaire on the availability of dietary assessment methods was developed and results from eighteen African countries were presented [9]. In contrast to Europe, the inventory showed that for the African continent, high quality, validated and standardized tools are currently lacking, making it difficult to monitor the different phases and speed of the nutrition transition across its countries. Due to this in depth inventory published in the framework of the AS-PADAM project, it was decided to exclude Africa as well from this cross-continental inventory.

As mentioned before, in light of comparability of results cross-continentally, a thorough overview and comparison of methodological aspects associated with these surveys in each continent is requested. Therefore, the aims of the present paper are (1) to develop a framework of key parameters describing methodological aspects of FCS, (2) to create an inventory of methodological properties of national food consumption surveys performed on the continents North-America, South-America, Asia and Australasia, and the remaining continents for which such in depth inventory is still missing, and (3) to discuss and compare these methodological properties cross-continentally.

## 2. Experimental Section

### 2.1. Development of the Inventory Framework

First, key methodological properties of FCS were identified in order to construct a framework available for developing the inventory. This framework was based on the one used by Huybrechts and co-workers [8]. After author debate, it was decided to categorize the properties into six aspects of conducting an FCS: (1) target population, survey design and sampling, (2) dietary intake and other assessments, (3) recruitment of participants, (4) fieldwork characteristics, (5) data/nutrient analyses, and (6) recruitment and training of the interviewers. The framework was designed as a table listing FCS in the rows and property fields in the columns. In total, twenty-nine fields were created. The fields to be completed per survey are presented in Table 1.

**Table 1:** Overview of inventory framework.

**General items**	**Recruitment of participants**	**Recruitment and training of interviewers**
Continent	Invitation type	Recruitment criteria interviewers
Country	Incentives	Number of interviewers
Survey	Number of participants (*n*)	Training material/Training topics
**Target population, survey design and sampling**	Participation rate (%)	Training duration
Sex	Problems in recruitment	
Age (years)	**Fieldwork characteristics and data controls**	
Sampling method and design	Place of DIA administration	
Sampling frame	Time-span fieldwork	
**Dietary intake and other assessments**	Intermediate controls	
Method	Final data controls	
Total recalls (*n*)	**Food linking and analysis**	
Administration	Food classification system	
Portion size estimation	Food composition databases	
Interview aids/software	Statistical procedures/ adjustment (software)	
Measured anthropometrics	Methods for calculating under- or overreporters	
Biological samples		

DIA: dietary intake assessment.

### 2.2. Search Strategy

As proposed by Blanquer *et al*. [10], a combined strategy for data acquisition was used. Firstly, a systematic literature search was performed and subsequently, experts were contacted to complete missing information which could not be found in the literature. We used the electronic database MEDLINE (PubMed) and Web of Science to identify studies reporting on food consumption surveys from 1985 to December 2011. Text terms with appropriate truncations, Boolean operators and relevant indexing terms were used. The reference lists in the articles, reviews and textbooks retrieved were also investigated for additional publications yielding a substantial amount of grey literature like reports available on websites of governmental bodies. The key words used in the search were: “national nutrition survey”; “food and nutrition survey”; “dietary consumption survey”; “dietary intake”; “nutrition examination”; “nutrition survey”; and “dietary intake assessment”. Additional terms referring to a country or continent were added to this search query for obtaining region-specific information. The selection of continents was based on the seven-continent model excluding Europe (pan-European methodology and inventory of experiences are reported elsewhere [7,8,11]), Africa (availability of dietary assessment tools in Africa have been reported previously by Gavrieli *et al*. [7]) and Antarctica (no permanent habitation).

The exclusion criteria that were used to withdraw retrieved surveys were: (1) age (nutrition surveys in children only were excluded given their age-specific approach in terms of dietary intake assessment); (2) indirect or ecological measurement of food intake (e.g., food balance sheets or household budget surveys); (3) absence of dietary intake assessment (e.g., nutritional assessment based on anthropometric or clinical measurements), and (4) publications or reports not available in English and/or not accessible online.

Once the table was completed based on the information available from the retrieved publications, it was e-mailed to principal investigators or corresponding authors of studies reporting on the food consumption survey with an accompanying request to fill in the blanks. This additional information was then merged with the tables and the inventory was distributed to all collaborators for final review.

## 3. Results

The first step of the search strategy yielded a total of 12,605 articles. From this, 4,511 articles met at least one of the exclusion criteria. In the remaining articles, single surveys from individual countries were identified. A total of ten countries from four continents were retained: North-America: Canada, United States (US), Mexico; South-America: Brazil; Asia: China, Japan, Korea (South), Malaysia; Australasia: Australia, New Zealand. In total, data from 28 FCS are presented in the overview.

### 3.1. Target Population, Survey Design and Sampling Method

Table 2 summarizes the study design aspects and methods of the selected surveys. The ages of the target populations ranged from less than 1 year of age to over 80 years. Surveys including all age categories were from Canada, US, Mexico (MHNS-06), China (1991 and onwards), Japan, Korea and Australia. In all surveys, both genders were included except for Mexico (NNS-1999) that included women only. In all surveys, a multistage sampling design was used to select study participants. The sampling frames used for selection of sampling units were based either on census data (US, Mexico, Brazil, Korea and New Zealand), a combination of frames like healthcare registries and labour force data (Canada), strata from counties (China), or enumeration blocks (geographical areas which are artificially created to have about 80 to 120 living quarters (Malaysia)). For Canada, the US, Mexico, China, Korea and Australia the national food consumption survey was also part of a health (examination) survey. The dietary monitoring surveys were cross-sectional, some of which have a continuing character since they are repeated annually or biennially (the US, China, Japan and Korea). For the US and China, participants are included in a cohort for tracking over time.

### 3.2. Numbers of Participants and Participation Rates

In Table 3, recruitment aspects of all selected surveys are listed. Sample sizes of single surveys ranged from 2,596 (Mexico; NNS-1999) to over 30,000 (Canada and Brazil). This latter figure was larger when taking into account the totals of all samples in the continuous programs in the US, China and Korea. Participation rates were above 90% in Korea (KNHANES 1998) and Malaysia; between 80.0%–89.9% in the US (NHANES 2001, 2005), Mexico (NNS-1999), Brazil, China and Korea; between 70.0%–79.9% in Canada, the US (NHANES 2003, 2007 and 2009), and Australia (for the FFQ); and below 70% in Japan, Australia (for the 24-HDR) and New Zealand.

**Table 2:** Target population, survey design and sampling method of national nutrition surveys per continent.

Continent *Country* *[Ref.]*	Survey name	Institution	Year(s)	Sex	Age (years)	Sampling method and design	Sampling frame
North-America							
*Canada* *[12,13]*	Canadian Community Health Survey - Nutrition (CCHS)	Statistics Canada	2004	M and F	All age categories (<1–71+)	Two-step strategy: 1) 80 units in 14 age/sex groups per province 2) power allocation scheme for remaining anticipated units	4 frames: Labour Force Survey (LFS) area frame, CCHS 2.1 dwellings, Prince Edward Island and Manitoba Healthcare registries
*US* *[14,15]*	What we Eat in America (WWEIA), National Health and Nutrition Examination Survey (Continuous NHANES)	National Center for Health Statistics (NCHS) from the Centers for Disease Control and Prevention (CDC)	2001–2002	M and F	All age categories (< 1–80+)	Stratified, multistage probability sample: Primary Sampling Units (PSUs) (counties) > segments within PSUs (blocks containing a cluster of households) > households within segments > one or more participants within households	PSU samples were selected from a frame of all U.S. counties, using the 2000 census data and associated estimates and projections
			2003–2004	〃	〃	〃	〃
			2005–2006	〃	〃	〃	〃
			2007–2008	〃	〃	〃	〃
			2009–2010	〃	〃	〃	〃
*Mexico* *[16,17,18,19,20]*	National Nutrition Survey 1999 (NNS-1999)	Instituto Nacional de Salud Pública (INSP)	1998–1999	Adolescents and adults: F Children: M and F	12–49 <12	Probabilistic, multistage, stratified cluster sample: basic geographical statistical area (BGSA) > household block > household	Census data (1995), stratification of BGSA by socioeconomic status index
	Encuesta Nacional de Salud y Nutrición 2006 (ENSANUT 2006), Mexican Health and Nutrition Survey 2006 (MHNS-06)	Instituto Nacional de Salud Pública (INSP)	2005–2006	Children: M and F Adults: M and F	<19 ≥19	Multistage, stratified cluster sample	n/a
South-America							
*Brazil* *[21]*	Brazilian Individual Dietary Survey (IDS 2008-2009)	Instituto Brasileiro de Geografia e Estatistica (IBGE)	2008–2009	M and F	≥10	Probabilistic two-stage complex cluster sampling: census tracts > households	Census data (2000), a subsample (25%) of households selected in the Household Budget Survey was randomly selected to participate in the IDS
Asia							
*China* *[22,23]*	China Health and Nutrition Survey (CHNS)	National Institute of Nutrition and Food Safety (NINFS) from the China Center for Disease Control and Prevention (CCDC)	1989	Children: M and F Adults: M and F	1–6 20–45	Multistage, random cluster sample: province > county > PSUs (*n* = 190) > household	Stratification of counties by income (low, middle, and high), four counties per province were selected, PSUs are urban neighborhoods, suburban neighborhoods, towns, and rural villages
			1991	M and F	All age categories	〃	〃
			1993	〃	〃	〃	〃
			1997	〃	〃	〃	〃
			2000	〃	〃	Multistage, random cluster sample: province > county > PSUs (*n* = 216) > household	〃
			2004	〃	〃	〃	〃
			2006	〃	〃	〃	〃
			2009	〃	〃	〃	〃
*Japan* *[24,25]*	National Nutrition Survey in Japan (NNS-J)	National Institute of Health and Nutrition (NIHN)	2004–2007	M and F	≥1−70+	Stratified random sample:survey district units (*n* = 300) > households	n/a
*Korea* *[26,27]*	Korean National Health and Nutrition Examination Survey (KNHANES)	Korean Institute for Health and Social Affairs (KIHASA) and the Korea Health Industry Development Institute (KHIDI)	1998	M and F	≥1 − 70+	Stratified, multistage probability sample: PSUs (*n* = 600) > households	Census data, population register
		〃	2001	〃	〃	〃	〃
		KIHASA, KHIDI and the Korean Centers for Disease Control and Prevention (KCDC)	2005	〃	〃	〃	〃
		KCDC	2007	〃	〃	〃	〃
		〃	2008	〃	〃	〃	〃
		〃	2009	〃	〃	〃	〃
*Malaysia* *[28,29]*	Malaysian Adult Nutrition Survey (MANS)	Ministry of Health Malaysia (MOH-M)	2004	M and F	18–59	Stratified random sample with proportional allocation	Enumeration Blocks (EB) and Living Quarters (LQ) were sampled proportionate to population size
Australasia							
*Australia* *[30,31,32,33]*	National Nutrition Survey (NNS)	Australian Bureau of Statistics (ABS) and Commonwealth Department of Health and Family Services (HFS)	1995	M and F	≥ 2	Multistage, area-based sample	Householders in private dwellings in 8 states and territories; Area-based selection using census collector districts from the 1991 Population Census
*New Zealand* *[34,35,36]*	New Zealand National Nutrition Survey (NNS97)	New Zealand Ministry of Health (MOH-NZ)	1996–1997	M and F	≥ 15	Multistage, stratified sample: PSUs (*n* = 18,000) > households > participant	Area based, census data (1991)
	New Zealand Adult Nutrition Survey (NZANS)	〃	2008–2009	〃	〃	Multistage, stratified, probability-proportional-to-size (PPS) sample	Area based, New Zealand census meshblocks (2006)

M: male; F: female; 〃: ditto; n/a: not available

**Table 3:** Dietary intake and other assessments of national nutrition surveys per continent.

Continent			Dietary intake assessment						
*Country* *[Ref.]*	Survey name	Year(s)	Method	Total recalls (*n*)	Administration of method	Portion size estimation	Interview aids/software	Measured anthropometrics	Biological samples
North-America									
*Canada* *[12,13]*	Canadian Community Health Survey - Nutrition (CCHS)	2004	24-HDR (children: 6-11 years assisted by parents; <6 years reported by parents)/ FFQ (past year, fruit and vegetables only)	1 (70% of sample) 2 (30% of sample)	Face-to-face (first interview) Telephone (recall)/ Paper-pencil	Food model booklet, volume measures (tablespoon, cup, etc.), weight measures (ounce, gram, etc.), dimensions (length, width, etc.), general measures (relative sizes, container units)	CAI software, developed by Statistics Canada (adopted from AMPM, USDA)	Weight and height	n/a
*US* *[14,15]*	What we Eat in America (WWEIA), National Health and Nutrition Examination Survey (Continuous NHANES)	2001–2002	24-HDR (children < 16 years proxy provided information)/ FFQ (past year, 124 items)	1	Face-to-face/ Paper-pencil	Three-dimensional food models for first interview.	CAI software, developed by USDA: Automated Multiple-Pass Method (AMPM)	Body composition and bone density (Dual energy x-ray absorptiometry), body measurements.	For a complete list of laboratory components of NHANES 1999–2012 visit http://www.cdc.gov/nchs/nhanes/about_nhanes.htm.
		2003–2004	〃	2 (3–10 day interval)	Face-to-face (first interview) Telephone (recall)	Three-dimensional food models for first interview. USDA’s Food Model Booklet (two-dimensional drawings of glasses, mugs, bowls, mounds, circles, *etc.*) and three-dimensional models (measuring cups and spoons, a ruler, and two household spoons) for telephone interview.	〃	〃	〃
		2005–2006	〃	〃	〃	〃	〃	〃	〃
		2007–2008	〃	〃	〃	〃	〃	〃	〃
		2009–2010	〃	〃	〃	〃	〃	〃	〃
*Mexico* *[16,17,18,19,20]*	National Nutrition Survey 1999 (NNS-1999)	1998–1999	24-HDR	1	n/a	n/a	n/a	Weight and height (in women, waist and hip circumferences)	Capillary blood: concentration of hemoglobin Venous blood and urine: assessment of micronutrient status
	Encuesta Nacional de Salud y Nutrición 2006 (ENSANUT 2006), Mexican Health and Nutrition Survey 2006 (MHNS-06)	2005–2006	Semi-quantitative FFQ (past 7 days, 101 foods, 14 food groups)		n/a	n/a	n/a		
South-America									
*Brazil* *[21]*	Brazilian Individual Dietary Survey (IDS 2008-2009)	2008–2009	2-day EDR (non-consecutive on pre-determined days spanning one week)		Paper pencil, face-to-face interview to review food records	Picture book (pictures of plates, glasses, bottles and cutlery)	CAPI software	Weight and height	n/a
Asia									
*China* *[22,23]*	China Health and Nutrition Survey (CHNS)	1989	24-HDR (children < 12 years proxy provided information)	3 (consecutive on pre-determined days spanning one week)	Paper pencil, face-to-face interview	Food models and picture aids	n/a	Weight and height, head circumference, arm circumference, and waist-hip ratio	None
		1991	〃	〃	〃	〃	〃	〃	〃
		1993	〃	〃	〃	〃	〃	〃	〃
		1997	〃	〃	〃	〃	〃	〃	〃
		2000	〃	〃	〃	〃	〃	〃	〃
		2004	〃	〃	〃	〃	〃	〃	〃
		2006	〃	〃	〃	〃	〃	〃	〃
		2009	〃	〃	〃	〃	〃	〃	Blood collection
*Japan* *[24,25]*	National Nutrition Survey in Japan (NNS-J)	2004–2007	1- or 3-day semi-weighed DR/ FFQ (≥20 years/ past 2 months, 122 foods and composite dishes)		Paper pencil, face-to-face interview to review food records/ Paper-pencil	Kitchen scale	n/a	Weight and height (subjects aged 1 year or older), abdominal circumference (subjects aged 6 year or older)	Blood collection (subjects aged 20 years or older)
*Korea* *[26,27]*	Korean National Health and Nutrition Examination Survey (KNHANES)	1998	24-HDR (in 200 PSUs)/ FFQ (past year, 109 food items)	1	Face-to-face/ Paper-pencil	Three-dimensional food models and a picture book with color photographs of foods	n/a	Weight and height	Blood and urine collection
		2001	〃	〃	〃	〃	n/a	〃	〃
		2005	〃	〃	〃	〃	n/a	〃	〃
		2007	〃	〃	〃	〃	n/a	〃	〃
		2008	〃	〃	〃	〃	n/a	〃	〃
		2009	〃	〃	〃	〃	n/a	〃	〃
*Malaysia* *[28,29]*	Malaysian Adult Nutrition Survey (MANS)	2004	24-HDR/ FFQ (past year, 126 foods, 15 food groups)	1	Face-to-face/ Paper-pencil	Album of food pictures and household measures	Nutritionist Pro™ Nutrition Analysis Software (for data entry)	Weight and height	n/a
Australasia									
*Australia* *[30,31,32,33]*	National Nutrition Survey (NNS)	1995	24-HDR (children: 2-4 years reported by adult; 5-11 yrs assisted by adult)/ FFQ (≥ 12 years/ past year, 107 foods)	1 (90% of sample)2 (10% of sample)	Face-to-face/ Paper-pencil	Measuring cups and spoons, grids and ruler	Food instruction booklet with types of foods and quantities of 15 food groups	Weight and height, waist and hip circumference	n/a
*New Zealand* *[34,35,36]*	New Zealand National Nutrition Survey (NNS97)	1996–1997	24-HDR/ FFQ (past year, 9 food categories)	1 2 (*n* = 695)	Face-to-face/ Paper-pencil	Cups, spoons, thickness sticks (thickness of meat, fish, poultry and cheese), photographs , grids and concentric circles, balls (to estimate apples and oranges), beans bags (to describe mashed potato and rice), standard serving sizes of foods and weights	CAPI software, LINZ24© (analogous to AMPM, USDA)	Weight and height, circumference of waist, hip and arm, waist-hip ratio, triceps and subscapular skinfold thickness, elbow breadth	Non-fasting blood sample: cellular evaluation, blood lipids, iron
	New Zealand Adult Nutrition Survey (NZANS)	2008–2009	24-HDR/ dietary habits questionnaire	1 (75% of sample) 2 (25% of sample)	Face-to-face/ Paper-pencil	Food photographs, shape dimensions, food portion assessment aids (e.g. dried beans) and packaging information	〃	Weight and height, waist circumference	Non-fasting blood sample: cellular evaluation, blood lipids, iron, HbA1c Spot urine sample: sodium, potassium, iodine, creatinine

〃: ditto; n/a: not available; EDR: Estimated dietary record; CAI: computer assisted interview; CAPI: computer assisted personal interview; AMPM: Automated Multiple-Pass Method.

### 3.3. Dietary Intake Assessment Methods

Most surveys used 24-HDR as the principal DIA method (Table 4). Multiple recalls for all participants were available in the US (2 recalls in NHANES 2003 and onwards) and China (3 recalls). In some countries, duplicate recalls were available in a subsample only (Canada, Korea, Australia and New Zealand). A computer-assisted personal interview (CAPI) was performed in the US (NHANES 2001), Malaysia and New Zealand. In Canada and the US (NHANES 2003 and onwards), a CAPI was performed during the first recall and a computer assisted telephone interview (CATI) during the second recall. In the surveys from China and Australia, the 24-HDR was performed with paper and pencil in a face-to-face interview. In Korea, a face-to-face interview was performed, no interview software was reported, and in Mexico, the administration of the 24-HDR was also not reported in the study report. A prospective DIA method was only used in Brazil and Japan (2-day EDR and 1- or 3-day semi-weighed DR respectively). Finally, Mexico (MHNS-06) used only a semi-quantitative FFQ to report on frequencies of intake during the past seven days. An FFQ (formerly called Food Propensity Questionnaire) was also used in addition to a principal DIA method to identify frequencies of consumption and non-consumers of various food groups in Canada, the US, Japan, Korea, Malaysia, Australia and New Zealand (NNS97).

### 3.4. Fieldwork Characteristics and Data Controls

In Table 5, the fieldwork aspects of the nutrition surveys are presented. All surveys reported that at least one interview was conducted when the participant was at home. For surveys with multiple interviews, at least one was conducted at home. Interviews could either be a face-to-face or a telephone interview. In cases where the DIA was a dietary record, interviews were performed to review the participant’s records and to check for completeness (Brazil and Japan). Another place for administrating the DIA was at mobile examination centres (MEC) (the US, NHANES). The time-span of the fieldwork was at least one year (all seasons) in Canada, the US, Brazil, Korea (KNHANES 2008 and onwards), Malaysia, Australia and New Zealand.

### 3.5. Food Linking and Analysis

Table 6 summarizes features related to data analyses of the nutrition surveys. Surveys using multiple measures of intake are able to correct for within-person variability. Most surveys used the Nusser method (using Software for Intake Distribution Estimation SIDE or C-SIDE) developed at the Iowa State University (ISU) to calculate distributions of usual intake (Canada, US NHANES 2003, Brazil, Korea and New Zealand). For the US, from NHANES 2005 and onwards, the NCI method developed by the National Cancer Institute was used. Finally, in the Australian survey, an equation by the US National Academy of Science (NAS) was used to adjust for within-person variance [33]. Furthermore, misreporting of energy intake was assessed using either the Goldberg method [37] (EI:BMR_est_) (the US, Brazil, Malaysia and Australia) or the equations by Black and Cole [38] (Canada). Two surveys indicated that no calculation of misreporting was performed (Korea and New Zealand).

**Table 4:** Recruitment of the participants in national nutrition surveys per continent.

Continent							
*Country* *[Ref.]*	Survey name	Year(s)	Invitation type	Incentives	Number of participants (*n*)	Participation rate (%)	Problems in recruitment/ recruitment notes
North-America							
*Canada* *[12,13]*	Canadian Community Health Survey-Nutrition (CCHS)	2004	Invitation letter and telephone invitation	None	35.107	76.5	Difficulties in approaching target population, participation was experienced as burdensome
*US* *[14,15]*	What we Eat in America (WWEIA), National Health and Nutrition Examination Survey (Continuous NHANES)	2001–2002	Invitation letter, personal visit at home	Participants receive remuneration as well as reimbursement for transportation and child/elder care expenses	11.039	84.0	NHANES is designed to sample larger numbers of certain subgroups of particular public health interest. Oversampling is done to increase the reliability and precision of estimates of health status indicators for these population subgroups.
		2003–2004	〃	〃	10.122	79.0	〃
		2005–2006	〃	〃	10.348	80.5	〃
		2007–2008	〃	〃	10.149	78.4	〃
		2009–2010	〃	〃	10.537	79.4	〃
*Mexico* *[16,17,18,19,20]*	National Nutrition Survey 1999 (NNS-1999)	1998–1999	n/a	n/a	Adolescent F: 416 Adult F: 2,596	82.4	n/a
	Encuesta Nacional de Salud y Nutrición 2006 (ENSANUT 2006), Mexican Health and Nutrition Survey 2006 (MHNS-06)	2005–2006	n/a	n/a	Adolescents: 7,464 Adults: 21,113	n/a	n/a
South-America							
*Brazil* *[21]*	Brazilian Individual Dietary Survey (IDS 2008-2009)	2008–2009	Personal visit at home	None	34.032	81.0	The burden of participating in a survey was reported as a recruitment problem
Asia							
*China* *[22,23]*	China Health and Nutrition Survey (CHNS)	1989	Personal visit at home	n/a	15.927	n/a	Participants leaving in one survey and moving back in a later year, migration of participants, natural disasters and major redevelopment of housing in all large urban centres
		1991	〃	〃	14.789	88.1	〃
		1993	〃	〃	13.893	88.2	〃
		1997	〃	〃	15.874	80.9	〃
		2000	〃	〃	17.054	83.0	〃
		2004	〃	〃	16.129	80.2	〃
		2006	〃	〃	18.764	88.0	〃
		2009	〃	〃	n/a	n/a	〃
*Japan* *[24,25]*	National Nutrition Survey in Japan (NNS-J)	2004–2007	n/a	n/a	8,762 (2004) 8,885 (2007)	≈60.0 (a)	n/a
*Korea* *[26,27]*	Korean National Health and Nutrition Examination Survey (KNHANES)	1998	Invitation letter	Small present	11.525	95.9	n/a
		2001	〃	〃	10.051	81.0
		2005	〃	Small present and a letter with individual results from examination	9.047	80.5	The burden of participating in a survey and motivation of participants were reported as recruitment problems
		2007	〃	〃	4.099	80.6	〃
		2008	〃	〃	8.641	82.0	〃
		2009	〃	〃	9.397	82.2	〃
*Malaysia* *[28,29]*	Malaysian Adult Nutrition Survey (MANS)	2004	n/a	n/a	6.886	93.6 (24-HDR) 92.0 (FFQ)	n/a
Australasia							
*Australia* *[30,31,32,33]*	National Nutrition Survey (NNS)	1995	Invitation letter	None	13.858	61.4 (24-HDR) 76.0 (FFQ)	n/a
*New Zealand* *[34,35,36]*	New Zealand National Nutrition Survey (NNS97)	1996–1997	Telephone invitation and/or personal visit at home	Small present	4.636	50.1	Participants of the Health Survey were asked if they would further consent to the Nutrition Survey which badly affected the response rate since added respondent burden and time lapse between both surveys
	New Zealand Adult Nutrition Survey (NZANS)	2008–2009	Personal visit at home	Grocery voucher (if blood collected) and a letter with individual results from examination	4.721	61.0	〃

F: female; 〃: ditto; n/a: not available

**Table 5:** Fieldwork characteristics and data controls of national nutrition surveys per continent.

Country [Ref.]	Survey name	Year(s)	Place of DIA administration	Time-span fieldwork	Intermediate controls	Final data controls
North-America						
Canada [12,13]	Canadian Community Health Survey-Nutrition (CCHS)	2004	Participant’s home	Jan 2004–Jan 2005	Quality control at data entry, checking completeness and accuracy of collected data, regular meetings to review the progress of fieldwork and interviewers.	Identification of extreme values of nutrients and food groups. Calculation of misreporting (see table 6).
US [14,15]	What we Eat in America (WWEIA), National Health and Nutrition Examination Survey (Continuous NHANES)	2001–2002	First interview: Mobile Examination Center (MEC)	Jan 2001–Dec 2002	The CAPI software program has built-in data edit and consistency checks to reduce data entry errors. Interviewers were alerted the when unusual or potentially erroneous data values were recorded.	Interview records were reviewed by the NHANES field office staff for accuracy and completeness. A subset of the household interviews was verified by re-contacting the survey participants. Periodically, interviews were audio-taped and reviewed by NCHS and contractor staff.
		2003–2004	First interview: MEC Second interview: participant's home	Jan 2003–Dec 2004	〃	〃
		2005–2006	〃	Jan 2005–Dec 2006	〃	〃
		2007–2008	〃	Jan 2007–Dec2008	〃	〃
		2009–2010	〃	Jan 2009–Dec2010	〃	〃
Mexico [16,17,18,19,20]	National Nutrition Survey 1999 (NNS-1999)	1998–1999	n/a	Oct 1998–Mar1999	n/a	n/a
	Encuesta Nacional de Salud y Nutrición 2006 (ENSANUT 2006), Mexican Health and Nutrition Survey 2006 (MHNS-06)	2005–2006	n/a	Oct 2005–May 2006	n/a	n/a
South-America						
Brazil [21]	Brazilian Individual Dietary Survey (IDS 2008–2009)	2008–2009	Participant's home	May 2008–May2009	Cross-check data, quality control during data entry, completeness and accuracy checks of collected data, regular meetings to review the progress of fieldwork and make adjustments as required	Calculation of misreporting (see table 6).
Asia						
China [22,23]	China Health and Nutrition Survey (CHNS)	1989	Participant’s home	n/a	Internal controls on quality measures have been based on collecting measures of selected factors from multiple perspectives and then using these data to refine measurements.	Individual's average daily dietary intake, calculated from the household survey, was compared with dietary intake based on 24-h recall data. In case of discrepancies, households were revisited.
		1991	〃	〃	〃	〃
		1993	〃	〃	〃	〃
		1997	〃	〃	〃	〃
		2000	〃	〃	〃	〃
		2004	〃	〃	〃	〃
		2006	〃	〃	〃	〃
		2009		〃	〃	〃
Japan [24,25]	National Nutrition Survey in Japan (NNS-J)	2004–2007	Participant's home	n/a	Interview with participant to review food records and check for completeness	n/a
Korea [26,27]	Korean National Health and Nutrition Examination Survey (KNHANES)	1998	Participant’s home	Nov 1998–Dec 1998	Cross-check of data, participants were re-contacted to provide extra information when the data is incomplete or possibly wrong	Extreme values for some nutrients and food groups were calculated
		2001	〃	Nov 2001–Dec 2001	〃	〃
		2005	〃	Apr 2005–May2005	〃	〃
		2007	〃	Jul 2007–Dec 2007	〃	〃
		2008	〃	Jan 2008–Dec 2008	〃	〃
		2009	〃	Jan 2009–Dec 2009	〃	〃
Malaysia [28,29]	Malaysian Adult Nutrition Survey (MANS)	2004	Participant's home	Oct 2002–Dec 2003	Data entry clerks trained to identify, describe foods and recipes and performed quality control checks, interviewers reviewed the recall with the respondent to check for completeness and accuracy	Calculation of misreporting (see Table 6).
Australasia						
Australia [30,31,32,33]	National Nutrition Survey (NNS)	1995	Participant’s home	Feb 1995–Mar 1996	Data was checked immediately after collection using standardised checklists. During data entry, all data was scrutinized and quality control checks for extreme quantities were built-in to the data entry computer system.	Extreme values for for energy, macro-nutrients and micro-nutrients by age and sex were checked. Calculation of misreporting (see Table 6).
New Zealand [34,35,36]	New Zealand National Nutrition Survey (NNS97)	1996–1997	Participant’s home	Dec 1996–Nov 1997	Interviewers sent diet recalls to project office within 24 hours of collection so the project office could check each recall for accuracy and completeness which enabled interviewers to go back to participants, and/or clarify data with project office	Extreme values for nutrient intakes were scrutinised after conversion of food to nutrients
	New Zealand Adult Nutrition Survey (NZANS)	2008–2009	Participant’s home	Oct 2008–Oct 2009	〃	〃

〃: ditto; n/a: not available

**Table 6:** Food linking and analysis of national nutrition surveys per continent.

Continent						
*Country* *[Ref.]*	Survey name	Year(s)	Food classification system	Food composition databases	Statistical procedures/adjustment (software)	Methods for calculating under- or overreporting
North-America						
*Canada* *[12,13]*	Canadian Community Health Survey—Nutrition (CCHS)	2004	Bureau of Nutritional Sciences (BNS) food groups, based on British and American food group systems	Nutrition Survey System (NSS)	Nusser method using SIDE (Iowa State University)	Equations by Black and Cole
*US* *[14,15]*	What we Eat in America (WWEIA), National Health and Nutrition Examination Survey (Continuous NHANES)	2001*–*2002	Food Surveys Research Group (FSRG) defined food groups	USDA Food and Nutrient Database (FNDDS), 1.0	SUDAAN was used to adjust for survey design effects resulting from NHANES’ complex, multistage, probability sampling	Calculation of EI:BMRest
		2003*–*2004	〃	USDA Food and Nutrient Database (FNDDS), 2.0	Nusser method using C-SIDE (Iowa State University)	〃
		2005*–*2006	〃	USDA Food and Nutrient Database (FNDDS), 3.0	NCI method	〃
		2007*–*2008	〃	USDA Food and Nutrient Database (FNDDS), 4.1	〃	〃
		2009*–*2010	〃	USDA Food and Nutrient Database (FNDDS), 5.0	〃	〃
*Mexico* *[16,17,18,19,20]*	National Nutrition Survey 1999 (NNS-1999)	1998*–*1999	n/a	USDA Nutrient database for standard reference, University of California Food composition database, Tabla de composición de alimentos para uso en América Latina (PAHO, INCAP), Tablas de composición de alimentos mexicanos del Instituto Nacional de Ciencias Médicas y Nutrición Salvador Zubirán, Tablas de valor nutritivo de los alimentos de mayor consumo en México, Food composition and nutrition tables (Souci, Fachmann & Kraut)	n/a	n/a
	Encuesta Nacional de Salud y Nutrición 2006 (ENSANUT 2006), Mexican Health and Nutrition Survey 2006 (MHNS-06)	2005*–*2006	n/a	n/a	n/a	n/a
South-America						
*Brazil* *[21]*	Brazilian Individual Dietary Survey (IDS 2008–2009)	2008*–*2009	National food classification system	Nutrition Coordination Center Nutrient Databank (Nutrition Data System for Research—NDSR, Minneapolis), Brazilian Food Composition Table (TACO)	NCI method	Calculation of EI:BMRest
Asia						
*China* *[22,23]*	China Health and Nutrition Survey (CHNS)	1989	n/a	Food Composition Table for China (ed. 1991)	n/a	n/a
		1991	〃	〃	〃	〃
		1993	〃	〃	〃	〃
		1997	〃	〃	〃	〃
		2000	〃	〃	〃	〃
		2004	〃	Food Composition Table for China (ed. 2002)	〃	〃
		2006	〃	Food Composition Table for China (ed. 2004)	〃	〃
		2009	〃	〃	〃	〃
*Japan* *[24,25]*	National Nutrition Survey in Japan (NNS-J)	2004-2007	n/a	Standard Tables of Food Composition in Japan	n/a	n/a
*Korea* *[26,27]*	Korean National Health and Nutrition Examination Survey (KNHANES)	1998	National food classification system	Food composition table from the National Rural Living Science Institute	Nusser method using C-SIDE (Iowa State University)	Not applied
		2001	〃	〃	〃	〃
		2005	〃	〃	〃	〃
		2007	〃	〃	〃	〃
		2008	〃	〃	〃	〃
		2009	〃	〃	〃	〃
*Malaysia* *[28,29]*	Malaysian Adult Nutrition Survey (MANS)	2004	n/a	USDA Food Database, Canadian Food Database, Mexico Food Database, Malaysian Food Composition Tables (all available in Nutritionist Pro), Singapore Food Composition Guide, ASEAN Food Composition Tables, and The China Food Composition Tables	n/a	Calculation of EI:BMRest
Australasia						
*Australia* *[30,31,32,33]*	National Nutrition Survey (NNS)	1995	National food classification system developed by ANZFA	NNS nutrient composition database AUSNUT (1999) developed by the Australia New Zealand Food Authority (ANZFA). Food and beverage intake data were coded using the Australian Nutrition Survey System (ANSURS).	Adjustment for within-person variability using the equation put forward by the US National Academy of Science (NAS) Subcommittee on Criteria for Dietary Evaluation (1986)	Calculation of EI:BMRest
*New Zealand* *[34,35,36]*	New Zealand National Nutrition Survey (NNS97)	1996–1997	National food classification system	New Zealand Food Composition Database (NZFCD), FOODfiles electronic subset of data from the NZFCD, NUTTAB Food Composition Tables (Australia), McCance and Widdowson’s Composition of Foods and other international data as required	Nusser method using C-SIDE (Iowa State University)	Not applied
	New Zealand Adult Nutrition Survey (NZANS)	2008–2009	〃	〃	〃	〃

〃: ditto; n/a: not available.

**Table 7:** Recruitment and training of the interviewers in national nutrition surveys per continent.

Continent							
*Country* *[Ref.]*	Survey name	Year(s)	Recruitment criteria interviewers	Number of interviewers (*n*)	Training material/Training topics	Training duration	Remarks
North-America							
*Canada* *[12,13]*	Canadian Community Health Survey - Nutrition (CCHS)	2004	Professional interviewers who work on a variety of surveys, full-time and part-time	600	Software training, interview training	3, 5 days	
*US* *[14,15]*	What we Eat in America (WWEIA), National Health and Nutrition Examination Survey (Continuous NHANES)	2001–2002	High School diploma required/BA preferred	n/a	Intensive training course and supervised practice interviews, periodic and annual retraining sessions	2 weeks	
		2003–2004	〃	〃	〃	〃	
		2005–2006	〃	〃	〃	〃	
		2007–2008	〃	〃	〃	〃	
		2009–2010	〃	〃	〃	〃	
*Mexico* *[16,17,18,19,20]*	Mexican Health and Nutrition Survey 2006 (MHNS-06)	2005–2006	n/a	n/a	n/a	n/a	
	Encuesta Nacional de Salud y Nutrición 2006 (ENSANUT 2006), Mexican Health and Nutrition Survey 2006 (MHNS-06)	2005–2006	n/a	n/a	n/a	n/a	
South-America							
*Brazil* *[21]*	Brazilian Individual Dietary Survey (IDS 2008-2009)	2008–2009	n/a	n/a	Software training, training on contacting participants, interview training, data-collection skills	1 week	
Asia							
*China* *[22,23]*	China Health and Nutrition Survey (CHNS)	1989	Trained nutritionists	160	Specific training in the collection of dietary data for field staff and office staff	3 days	
		1991	〃	〃	〃	〃	
		1993	〃	〃	〃	〃	
		1997	〃	〃	〃	〃	
		2000	〃	〃	〃	〃	
		2004	〃	〃	〃	〃	
		2006	〃	〃	〃	〃	
		2009	〃	〃	〃	〃	
*Japan* *[24,25]*	National Nutrition Survey in Japan (NNS-J)	2004–2007	Registered dietitians and dietitians for nutrition component of health survey	n/a	n/a	n/a	
*Korea* *[26,27]*	Korean National Health and Nutrition Examination Survey (KNHANES)	1998	Trained dietitians/nutritionists	160	Training on contacting participants, interview training, data-collection skills	5 days	
		2001	〃	100	〃	3 days	
		2005	〃	150	〃	4 days	
		2007	〃	10	〃	11 days	A smaller number of well-trained dietitians were used after changing to the annual survey
		2008	〃	12	〃	10 days	
		2009	〃	12	〃	15 days	
*Malaysia* *[28,29]*	Malaysian Adult Nutrition Survey (MANS)	2004	Nutritionists familiar with local food customs	n/a	Training on interviewing and probing skills, quantification of portion sizes of foods	n/a	
Australasia							
*Australia* *[30,31,32,33]*	National Nutrition Survey (NNS)	1995	Qualified dietitians and nutritionists	n/a	Intensive training and supervision of interviewers to reduce non-sampling errors	2 weeks	
*New Zealand* *[34,35,36]*	New Zealand National Nutrition Survey (NNS97)	1996–1997	Trained interviewers familiar with local food customs passing an admission test	n/a (every interviewer was assisted by one assistant)	Software training, training on contacting participants, interview training, data-collection skills and training on the use of the survey tools.	Interviewer: 2 weeks Assistant: 2 days	Additional training was provided at the regional level every two months. Pacific interviewers and assistants were trained to survey non-English speaking Pacific and Asian immigrant groups.
	New Zealand Adult Nutrition Survey (NZANS)	2008–2009	〃	22	〃	2 weeks	Additional training was provided at the regional level every three months. Pacific interviewers and assistants were trained to survey non-English speaking Pacific and Asian immigrant groups.

### 3.6. Recruitment and Training of Field Staff

In Table 7, recruitment and training of the interviewers and field staff in the nutrition surveys are listed. In China, Japan, Korea, Malaysia and Australia, it was mandatory that the interviewers be nutritionists or dietitians. In other countries, interviews were performed by trained interviewers, who were familiar with local food customs (New Zealand), or professional interviewers working on a variety of surveys (Canada). For interviewers in the US, a high school diploma was considered to be the minimum education requirement, as this is necessary for government jobs. Training was provided on a variety of topics like interviewing (and probing) skills (Canada, the US, Brazil, China, Korea, Malaysia, Australia and New Zealand), training on contacting participants, and software training. The duration of these training sessions ranged from three days (China) to fifteen days (Korea, KNHANES 2009). The average duration of reported training programs for interviewers was around seven days.

## 4. Discussion

This review presents an inventory of methodological aspects related to the performance of national food consumption surveys in different continents for which an in depth inventory on the dietary intake assessment methods used was still missing. Inventories covering both standardized and non-standardized data collection protocols and tools for capturing food consumption information on the European and African continent have been published before [7,8,9]. The present inventory comprises a total of twenty-eight food consumption surveys performed in ten countries from four continents: North-America, South-America, Asia and Australasia. In six countries (Canada, the US, Mexico, China, Korea and Australia), the FCS was part of a larger health examination survey from which three (the US, China and Korea) have been continuous programs. When surveys were not part of a larger health examination survey, the overview shows that questionnaires on health and physical activity were often still included.

The most common approach to assess dietary intake was the use of replicate 24-HDR in combination with an FFQ. In most countries, replicate 24-HDR interviews were administered to subsamples ranging from <10% to 30% of the total sample. For instance, in 2002, the Korean National Nutrition Survey by Season (KNNSS) was conducted and an additional 24-HDR was administered to a subsample of KNHANES over three subsequent seasons to offset seasonal variation in food intake [27]. Duplicate and triplicate 24-HDR were administered to all participants in the US and China respectively. A single 24-HDR without additional FFQ was used in Mexico (NNS-1999). In the more recent Mexican Health and Nutrition survey (MHNS-06), the 24-HDR was replaced by a semi-quantitative FFQ that was used to assess frequencies of consumption during the past seven days [17]. This FFQ included the 95% most consumed foods reported in the 24-HDR collected in the previous survey (MNS-99) [16]. Two countries used a dietary record to assess intakes (Brazil and Japan). However, a research group under the auspices of the Japanese Ministry of Health, Labour and Welfare suggested transferring the method currently in use from a semi-weighed dietary record combined with an FFQ to the 24-HDR making international comparisons possible [25]. Regardless of the DIA methods used, administration took place most often in the participants’ homes, providing the major advantage for interviewers to verify food packages or household measures in their home if this could help them to obtain more detailed information. In a study performed by Huybrechts *et al*. [8], participants of the EFCOVAL project were asked to indicate their preferred location for a future 24-HDR interview. Forty-nine percent of the subjects would prefer the study centre (*versus* 22% at home and 10% at work) if the interview was face-to-face and 63% would prefer to be at home for a telephone interview (compared with 11% at work). The high number of subjects that preferred the study centre for face-to-face interview might be explained because the EFCOVAL protocol required a visit to the study centre to collect blood samples and to provide participants with material for 24 h urine collections.

A large variety of portion size estimation tools was used in the different surveys ranging from three-dimensional aids like food models, cups, spoons and thickness sticks to two-dimensional albums or booklets depicting either photographs of foods, plates and glasses, or drawings of glasses, mugs and bowls (United States Department of Agriculture (USDA) food model booklet). The USDA Food Model Booklet was also adapted to create the USDA Food Models for Estimating Portions available for nutrition educators, consumers, and researchers to use outside of the context of the fully computerized Automated Multiple-Pass Method (AMPM) [39]. The AMPM is a validated five-step computerized dietary recall instrument developed by USDA and used in the “What We Eat in America” survey, the dietary intake interview component of the U.S. National Health and Nutrition Examination Survey (NHANES) [40,41]. Computer Assisted Interview (CAI) software is frequently used in national nutrition surveys because it allows structured and standardized collection of dietary intake data. The present overview shows that several countries use USDA-based CAI software and food classification. The leading role of this department is not surprising given its long history that goes back to 1892 [42]. Like North America, Europe has standardized its CAI software for future pan-European food consumption surveys [43]. The EPIC-Soft program, originally developed for the EPIC Study by the International Agency for research on Cancer (IARC), has been validated [44,45] and adapted to fit the purpose of pan-European food consumption surveys [46]. Recently, a name change of EPIC-Soft to GloboDiet software was announced, since this better suits the current and anticipated use of the increasingly widespread application of the tool worldwide [47].

Given that individual quantitative dietary intake surveys are expensive and difficult to implement, the Food and Agriculture Organization (FAO) Dietary Diversity questionnaire has been developed as a simple proxy to measure access to food at the household level [48] and micronutrient adequacy in women’s and children’s diets at the individual level [49,50].

Recruitment criteria for interviewers in national nutrition surveys are different between Asia and North America. In all Asian countries presented in the overview and Australia, interviews were conducted by either qualified/registered dietitians or nutritionists. In Japan, no interview was performed since dietary records were used; however, dietitians were recruited for data entry. In Canada and the US, it was not mandatory that the interviewers be dietitians or nutritionists. Both surveys rely either on professional interviewers involved in a variety of surveys or survey staff with a given minimal educational qualification, complemented with specific software and interview training. The duration of the training provided to interviewers varied across all available surveys from 2 days to 15 days (median duration: 7.5 days).

The current overview is the first of its kind to present a wide range of methodological aspects associated with national food consumption surveys across multiple continents. Although substantial efforts have been made to undertake a comprehensive overview, it is inevitable that some surveys were not captured. The present review qualifies as a narrative review and not a systematic review for a number of reasons. During the past decades, editors of scientific journals adopted reporting guidelines for producing systematic reviews. This was initiated in the medical research area enabling evidence-based decision making and improved health care. With the advent of these guidelines, publications on randomized (clinical) trials and intervention studies adhere to these criteria for inclusion in future systematic reviews. First, the time window of the present review including studies from 1985 exceeds the initiation of reporting guidelines by a decade so at that time, such guidelines were not yet available. Second, both guidelines for reporting as protocols to perform systematic reviews are not well adopted to studies using observational designs. Just recently, efforts have been made to adapt existing guidelines like the STROBE checklist (STrengthening the Reporting of OBservational studies in Epidemiology) to fit nutritional epidemiology studies (STROBE-NUT, reference equator). Third, a major source for information on methodological aspects of food consumptions surveys like details on sampling, instruments and training of staff are reports, information on websites of public agencies, both qualified as grey literature, and personal communications. These sources of information are sometimes not indexed in scientific databases and are, therefore, difficult to obtain using reproducible search strategies. Therefore, narrative reviews can be criticized because of their limited reproducibility. However, for reasons mentioned before, the two-step approach using both available literature and expert consultation, was the best method available to create the comprehensive overview presented.

This overview shows that the methods used for dietary intake assessment in national nutrition surveys are relatively similar across continents. The most frequently used method is the 24-HDR, sometimes administered repeatedly to correct for within-person variability, and mostly using interview software. Nevertheless, caution is still warranted when comparing results from food surveys between countries because of differences in conversion factors used for calculating nutrients (e.g., energy, protein, *etc*.). A variety of errors are introduced because many national or regional food composition tables or databases contain incomplete, outdated and unreliable data, or, countries borrow data from publicly available databases and neighbouring countries when such tables or databases are unavailable or inadequate [51].

Notwithstanding the growing consensus about the use of the 24-HDR methodology in food consumption surveys, the assessment remains self-reported. The most accurate and precise method for measuring energy expenditure is the doubly labeled water (DLW) method [52]. In weight stable conditions, one can expect that energy intake equals energy expenditure; hence, DLW is used in studies examining the validity of energy intake assessment. Such validation studies have indicated that the prevalence of energy underreporting in self-reported methods was about 30% (range: 12%–67%), and the magnitude of underestimation of energy intake was roughly 15% (range: 7%–20%) [53,54,55]. These reporting errors vary between men and women and are generally higher among overweight and obese subjects [41].

## 5. Conclusions

The 24-HDR was the most frequently used method in national food consumption surveys worldwide. Although this method is probably the most optimal to monitor dietary intakes of free-living subjects in large samples, it also has limitations and requires in depth training of the interviewers. In addition, future research is still necessary to explore and develop innovative methods that help us to measure dietary intake of populations and subgroups. For national FCS, it is recommended to combine different DIA methods like replicate 24-HDR and FFQs. For purposes of comparability of surveys, standardized procedures for data collection are required and a detailed description of the methods used should be included when reporting results. The inventory used in this review can serve as a guide to check if all methodological aspects related to the performance of a FCS are stated in such reports.
